# Sports Cardiology in Practice: Exercise as Stressor, Signal, and Therapy

**DOI:** 10.3390/jcdd13090432

**Published:** 2026-09-03

**Authors:** Christos Kourek, Eleftherios Karatzanos, Stavros Dimopoulos

**Affiliations:** 1 Clinical Ergospirometry, Exercise and Rehabilitation Laboratory, National and Kapodistrian University of Athens, 106 75 Athens, Greece; chris.kourek.92@gmail.com (C.K.); lkaratzanos@gmail.com (E.K.); 2 Department of Cardiac Surgery Research, Lankenau Institute for Medical Research, Wynnewood, PA 19096, USA; 3 Cardiac Surgery ICU, Onassis Cardiac Surgery Center, 176 74 Athens, Greece

Sports cardiology is often introduced as the discipline that decides whether an individual with a cardiovascular finding may exercise or compete. That description has become too narrow. In contemporary practice, the more difficult questions are how exercise modifies cardiovascular physiology over time, when an apparently physiological adaptation begins to carry clinical relevance, and how exercise itself should be prescribed when cardiovascular disease is already present. The second edition of the Special Issue “Sports Cardiology: From Diagnosis to Clinical Management” was developed around these questions. The eight contributions move from inherited arrhythmic disease and long-term electrical adaptation in athletes to autonomic regulation, cardiovascular rehabilitation, high-intensity interval training, inspiratory muscle training, hypertension, and functional aging [1,2,3,4,5,6,7,8]. The purpose of this closing Editorial is therefore to place these studies in a common clinical framework and to highlight what they collectively add to a field in which diagnosis, risk assessment, performance, and treatment increasingly overlap.

This evolution is also visible in recent recommendations. Contemporary sports cardiology no longer considers restriction the default response to every cardiovascular abnormality. The 2025 American Heart Association/American College of Cardiology scientific statement and the 2024 Heart Rhythm Society consensus both reinforce individualized risk assessment, shared decision-making, sport-specific evaluation, and return to activity whenever this can be achieved safely [9,10]. The 2020 European Society of Cardiology sports cardiology guidelines had already moved in the same direction, while prevention guidance continues to emphasize the broad health benefits of regular physical activity [11,12,13]. This change is important because the clinician is increasingly asked to distinguish among three very different situations: a physiological response to training, a cardiovascular substrate that becomes relevant under exercise, and a disease state in which exercise can itself be used therapeutically.

The review by Gonçalves et al. on Brugada syndrome (BrS) is a good example of how this distinction has changed clinical practice [3]. Historically, a diagnosis associated with sudden cardiac death often led directly to sports restriction. Yet the available evidence does not establish exercise as an independent predictor of events in BrS, and recommendations have consequently become less prohibitive. The authors emphasize the importance of symptoms, electrocardiographic phenotype, arrhythmic history, implantable cardioverter-defibrillator status, environmental exposure, and shared decision-making rather than diagnosis alone [3]. In asymptomatic individuals and genotype-positive/phenotype-negative subjects, competitive sport may be considered after appropriate evaluation, while hyperthermia, dehydration, electrolyte disturbances, and exercise under extreme environmental conditions remain clinically relevant concerns [14,15,16,17,18]. This approach is consistent with the wider shift in inherited arrhythmia management toward disease-specific risk assessment and exercise testing that reproduces the athlete’s actual physiological demands [9,10,11,19,20,21,22,23,24]. At the same time, the evidence base in BrS remains limited. The important message is therefore not that risk has disappeared, but that uncertainty should be managed through careful phenotyping and surveillance rather than automatic exclusion.

The study by Bondarev et al. approaches the electrical consequences of sport from the opposite direction: not the immediate risk of exercising with an inherited substrate, but the possibility that decades of training may leave a persistent electrophysiological footprint [4]. Among patients who received a pacemaker before the age of 70 years for otherwise idiopathic sinus-node or atrioventricular-node dysfunction, former athletes underwent implantation approximately five years earlier than non-athletes [4]. The authors appropriately avoid causal interpretation, given the retrospective design, small number of former athletes, and inevitable uncertainty surrounding lifetime training exposure. Nevertheless, the observation is clinically provocative. Athletic bradycardia has traditionally been attributed largely to increased vagal tone and considered reversible after detraining, but experimental and longitudinal data suggest that intrinsic sinus-node remodeling may also contribute [25,26,27,28]. Long-term follow-up studies in former endurance athletes and cross-country skiers have similarly raised the possibility of a higher burden of clinically relevant bradyarrhythmias in selected populations [25,26]. These findings should certainly not be used to discourage exercise, whose overall cardiovascular benefits are well established [12,13]. Rather, they remind us that “physiological” does not necessarily mean biologically neutral over an entire lifetime, particularly at very high cumulative training volumes.

The autonomic nervous system provides another bridge between adaptation and clinical interpretation. Oggionni et al. studied 117 elite male and female soccer players and used a composite Autonomic Nervous System Index (ANSI) derived from heart-rate variability parameters [5]. Male players had higher ANSI values than female players, and among men, midfielders and defenders, positions generally exposed to greater external aerobic load, showed higher values. In contrast, female players reported greater perceived stress and somatic symptoms, with the highest stress perception among midfielders and forwards [5]. These findings are relevant because the internal response to training is not fully captured by distance covered, speed, or session duration. Heart-rate variability has long been explored as a practical marker of training adaptation and recovery, but its interpretation is affected by age, sex, measurement conditions, and statistical methodology [29,30,31,32,33]. A composite index may simplify this complexity, although it cannot replace clinical assessment and requires validation in larger cohorts. The sex-specific findings also reinforce a broader need in sports cardiology: female athletes should not simply be evaluated by extrapolating physiological models developed predominantly in men.

Once exercise is treated as therapy rather than merely as exposure, the question of dose becomes central. This is particularly evident in cardiovascular rehabilitation, where exercise is strongly recommended but the effectiveness of a program depends on whether intensity, modality, progression, and adherence are sufficient to generate adaptation [34,35,36,37,38]. Two contributions to this Special Issue examine this problem from different perspectives. Gismondi et al. compared a repetitions-in-reserve rating of perceived exertion (RPE-RIR) approach with a percentage of estimated one-repetition maximum (%1RM) for resistance training in men with coronary artery disease undergoing rehabilitation [2]. Over nine weeks, the two approaches produced comparable strength gains, while the RPE-RIR strategy allowed training loads to respond to current performance rather than remain anchored to the initial assessment [2]. This concept of autoregulation is attractive in clinical populations, in whom sleep, medication, symptoms, nutrition, and day-to-day functional status may change the effective training stimulus. Resistance training has a growing evidence base in coronary disease and heart failure, and appropriate loading is necessary if the objective is to improve strength rather than merely complete a prescribed number of sessions [39,40,41,42]. The pilot nature and male-only sample require confirmation, but the study moves exercise prescription toward a more responsive, patient-specific model.

Evangelodimou et al. address another component of rehabilitation that is frequently less visible than limb-muscle training: the respiratory musculature [1]. Their systematic review of 15 studies found that inspiratory muscle training (IMT) before and/or after cardiac surgery can improve inspiratory muscle strength, pulmonary function, and functional capacity and may shorten intensive care unit stay [1]. Importantly, the available evidence did not establish a clear advantage of low- versus higher-intensity training, and only a limited number of studies initiated IMT preoperatively. This is clinically relevant because respiratory muscle weakness may already exist before surgery and can become more pronounced in the postoperative period. Previous systematic reviews and meta-analyses support the value of IMT around cardiac surgery, but heterogeneity in protocols, timing, and outcome assessment remains substantial [43,44,45]. Rather than searching for a single intensity that fits every patient, future work may be more useful if it identifies which patients benefit most, when training should begin, and how respiratory training should be integrated with early mobilization and conventional cardiac rehabilitation.

The review by Ko et al. broadens the discussion of exercise dose through high-intensity interval training (HIIT) [6]. Across 39 included studies, HIIT was associated with improvements in cardiorespiratory fitness, blood pressure, vascular function, lipid profile, body composition, and several measures of quality of life and mental health. The literature summarized by the authors also supports its application in selected higher-risk groups, including individuals with cardiovascular and metabolic disease [6]. Meta-analyses and randomized studies have shown that HIIT can improve peak oxygen uptake and vascular function and, in some settings, equal or exceed moderate-intensity continuous training despite shorter exercise sessions [46,47,48,49]. However, improvements in VO_2_peak should not automatically be equated with superior net clinical benefit. The incremental advantage of HIIT over moderate-intensity continuous training in cardiorespiratory fitness is often modest, whereas most trials are too small and too highly selected to determine differences in rare serious cardiovascular events. Vigorous exercise can transiently increase the risk of acute myocardial infarction or sudden cardiac death in susceptible individuals. In 4846 patients with coronary heart disease undergoing supervised cardiac rehabilitation, Rognmo et al. reported two nonfatal cardiac arrests during 46,364 h of HIIT and one fatal cardiac arrest during 129,456 h of moderate-intensity exercise [50]. A systematic review of 23 cardiac-rehabilitation studies found one major cardiovascular event during 17,083 HIIT sessions (11,333 training hours), suggesting a low absolute event rate in screened, clinically stable patients, but also emphasizing that the available safety evidence derives largely from supervised programs and remains insufficiently powered to exclude clinically important risk differences [51].

A separate issue is whether very high cumulative doses of high-intensity training can become biologically maladaptive. In healthy volunteers exposed to progressively increasing training loads, Flockhart et al. observed a marked reduction in intrinsic mitochondrial function accompanied by impaired glucose tolerance after the week with the highest exercise load [52]. Pataky and Nair highlighted these findings as a caution that excessive high-intensity exercise may adversely affect mitochondrial and metabolic homeostasis [53]. These observations should not be extrapolated directly to appropriately prescribed HIIT in cardiac rehabilitation, but they reinforce the concept that the dose–response relationship is not necessarily linear, and that greater intensity or training volume is not invariably better. VO_2_peak should therefore be viewed as an important efficacy endpoint rather than as a stand-alone “gold standard” for exercise-related health benefit. Adverse events, symptoms, ischemia or arrhythmia, hemodynamic tolerance, adherence, functional status, quality of life, and clinical outcomes should be considered alongside gains in cardiorespiratory fitness. Accordingly, “high intensity” should not be treated as a universal prescription. Protocols differ widely in interval duration, work-to-recovery ratio, total volume, and progression, and their practical value is greatest when patients are clinically stable, appropriately screened, and prescribed individualized intensity with supervised initiation and gradual progression when cardiovascular risk is increased [11,35].

Caminiti et al. examine the same issue in a much shorter exercise exposure: a single bout of isometric handgrip in patients with hypertension and ischemic heart disease [8]. In their randomized pilot study, both low- and high-intensity handgrip exercise were hemodynamically well tolerated, while high-intensity exercise at 70% of maximal voluntary contraction produced a significant reduction in systolic blood pressure during recovery compared with control, without evidence of impaired global myocardial work efficiency [8]. The result is noteworthy because isometric exercise has historically generated concern in coronary patients because of the possibility of excessive pressure loading. Current evidence increasingly recognizes isometric training as a potentially useful blood-pressure-lowering modality, although the magnitude of response depends on protocol and population [54,55,56,57]. The present study remains acute, relatively small, and restricted to stable, selected patients, so it should not be interpreted as a general endorsement of high-intensity isometric exercise in all patients with ischemic heart disease. It does, however, support further investigation of a time-efficient intervention that could be useful when conventional aerobic exercise is difficult to perform.

The contribution by Estévez-Caro et al. expands the scope still further by asking what cardiovascular disease means for everyday functional reserve in older adults [7]. Participants with hypertension performed worse than those without hypertension on several mobility and lower-limb performance measures, and the differences became particularly evident under dual-task conditions, in which a cognitive task was added to physical performance. Greater physical–cognitive interference was also observed in the hypertensive group [7]. This matters because clinical function in older adults is rarely a single-task activity. Walking while speaking, navigating an unfamiliar environment, carrying an object, or responding to an external stimulus requires cardiovascular, motor, and cognitive systems to operate simultaneously. Hypertension is closely linked to vascular aging and cognitive decline, and dual-task impairment may expose limitations not evident during conventional resting assessment [54,58,59,60]. From this perspective, exercise medicine is not only concerned with improving maximal performance. It is also concerned with preserving autonomy and reducing the trajectory toward frailty.

When these eight articles are read together, a consistent theme emerges [1,2,3,4,5,6,7,8]. Exercise acts as a stressor that may reveal latent disease, a biological signal capable of reshaping cardiovascular structure and electrophysiology, and a therapy whose effect depends on how it is prescribed. The same exercise stimulus can therefore have very different meanings according to phenotype, cumulative exposure, age, disease, and therapeutic objective. The athlete with BrS requires individualized arrhythmic risk assessment; the former endurance athlete with marked bradycardia may require longitudinal attention to conduction disease; the elite soccer player may benefit from interpretation of internal autonomic load alongside external workload; and the patient in rehabilitation may need exercise intensity adjusted to present capacity rather than a static baseline prescription. In older adults with hypertension, functional assessment may need to move beyond resting blood pressure and isolated strength testing toward tasks that better represent daily life.

The next step for sports cardiology is consequently not to accumulate more isolated exercise metrics, but to determine which measurements alter decisions. Prospective multicenter cohorts should quantify lifetime exercise exposure more accurately and establish whether specific thresholds of endurance training are associated with clinically meaningful electrical remodeling. Studies of inherited arrhythmias need event-based longitudinal data that can refine shared decision-making. Exercise-prescription trials should report intensity, progression, adherence, and adverse events in sufficient detail to make interventions reproducible, while women and older adults must be adequately represented. Dynamic phenotyping, through maximal exercise testing, ambulatory rhythm monitoring, autonomic assessment, functional dual-task testing, and advanced imaging, may ultimately be most useful when it is connected to a clear management consequence.

This second edition illustrates how far sports cardiology has moved from a simple question of sports eligibility. Its clinical territory now spans prevention, inherited disease, athlete physiology, rehabilitation, hypertension, aging, and the long-term consequences of exercise exposure. The contributions collected here do not support either unrestricted exercise or excessive caution. They support a more demanding approach: define the cardiovascular substrate, understand the exercise stimulus, measure the response that matters, and prescribe activity at a dose that is appropriate for the individual [1,2,3,4,5,6,7,8,9,10,11,12,13]. That is likely to be the most productive path toward preserving both cardiovascular safety and the considerable benefits of an active life.
